# Japanese Spotted Fever, South Korea

**DOI:** 10.3201/eid1207.051372

**Published:** 2006-07

**Authors:** Moon-Hyun Chung, Seung-Hyun Lee, Mi-Jeong Kim, Jung-Hee Lee, Eun-Sil Kim, Jin-Soo Lee, Mee-Kyung Kim, Mi-Yeoun Park, Jae-Seung Kang

**Affiliations:** * Inha University College of Medicine, Incheon, South Korea;; † Konkuk University College of Medicine, Chungju; South Korea;; ‡ National Institute of Health, Seoul, South Korea

**Keywords:** *Rickettsia japonica*, Japanese spotted fever, isolation, Korea

## Abstract

We describe the first case of Japanese spotted fever and the first isolate of spotted fever group rickettsia from a patient in South Korea. The isolated rickettsia from the patient was identified as *Rickettsia japonica* by analysis of the nucleotide sequences of 16S rRNA, *gltA*, *ompA*, *ompB*, and *sca4* genes.

The *Rickettsiaceae* family comprises obligate intracellular bacteria and contains 2 genera: *Rickettsia* (typhus group and spotted fever group[SFG]) and *Orientia*. Scrub typhus caused by *O. tsutsugamushi* is the most prevalent rickettsiosis in South Korea (*1*). SFG rickettsiae were first demonstrated to exist in South Korea by the isolation of *Rickettsia akari* from the Korean vole in 1957 (*2*). However, not a single case of rickettsialpox or other SFG rickettsiosis has been documented in Korea. Recently, evidence for the existence of SFG rickettsiosis has been provided by serologic survey and DNA detection in South Korea (*3**–**5*). Moreover, SFG rickettsiae displaying homology with *R. japonica* and *R. rickettsii* were detected in *Haemaphysalis* ticks by polymerase chain reaction analysis of the citrate synthase (*gltA*) gene, 16S rRNA, and *ompA* genes (*6*). However, no human cases of SFG rickettsiosis have been reported, and no SFG strain has been isolated from a person so far.

In this report, we present the first documentation of Japanese spotted fever in South Korea and isolation of *R. japonica*. To our knowledge, this is the first report of an SFG rickettsia isolated from a patient in South Korea.

## Case Report

**Figure 1:**
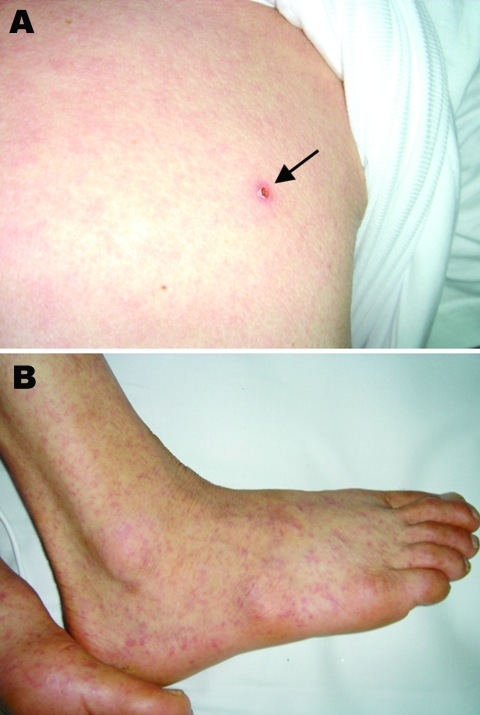
Skin findings of the patient. A small eschar (arrow) on the chest with erythematous rash (A) and petechiae (B) were observed.

A 65-year-old farmer was admitted to a hospital in Incheon, South Korea, on July 9, 2004; he had experienced fever, back pain, and myalgia for 5 days before admission. He lived in Mueui Island, ≈20 km east of Incheon. On physical examination, he had fever of 38.6°C, cervical and axillary lymphadenopathies, and a maculopapular rash. An eschar, which was smaller and more shallow than those of scrub typhus, was noticed on the chest wall (Figure 1). Laboratory studies showed a hemoglobin level of 7.7 mmol/L, a leukocyte count of 8 × 10^9^/L, and a platelet count of 87 × 10^9^/L. The patient was treated with oral doxycycline (200 mg/day), but the fever persisted during the treatment. On the third hospital day, petechiae developed on the trunk and extremities, including palms and soles (Figure 1). The leukocyte count increased to 11.2 × 10^9^/L, and the platelet count decreased further to 32 × 10^9^/L. He also showed confusion, irritability, and radiographic evidence of interstitial pneumonitis. The patient was then given azithromycin (500 mg/day intravenously) instead of oral doxycycline because of the possibility that he was infected with doxycycline-resistant *O. tsutsugamushi*. His fever resolved during next 5 days and he was discharged.

The serum samples were tested for antibody against *O. tsutsugamushi* (Boryong), *R. typhi* (Wilmington), and the isolated strain (Inha1) by using the indirect fluorescent-antibody (IFA) test. The serum specimen taken on the day of admission was negative for antibodies against all *Rickettsia* spp. by the IFA test. The serum sample taken after 18 days was sent to the national reference laboratory (Division of Rickettsial Diseases, Department of Bacteriology, National Institute of Health, Seoul, Republic of Korea) and was reported to be positive against *R. japonica* with a titer of 1,024. The convalescent-phase serum sample, taken on the 30th day after admission, was positive for antibodies against the isolated strain (Inha1) with a titer of 5,120.

To isolate the pathogen, a few drops of blood taken on the day of admission were added directly to the monolayer cultures of ECV304 cells, a spontaneously transformed cell line derived from human umbilical vein endothelial cells (obtained from S.Y. Kim) (*7*). After incubation for 24 h, monolayers of ECV304 were maintained in M199 medium containing 10% fetal calf serum and observed daily with an inverted microscope. The infected cells exhibited few cytopathologic changes, displaying only a few rounded cells. On day 28, IFA staining was done by using the convalescent-phase serum of the patient to visualize the rickettsiae. Many intracellular bacteria were observed inside the cells; they were not seen in the staining with control serum. From these results, we tentatively identified our isolated bacterium (strain Inha1), as a member of SFG rickettsiae.

**Figure 2:**
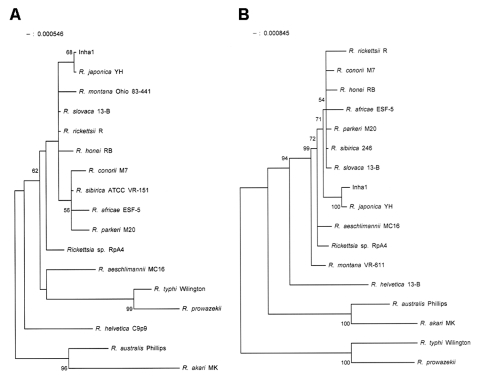
Phylogenetic tree based on 16S rRNA gene sequences (A) and *gltA* gene sequences (B) of the isolated rickettsial strain (Inha1). The phylogenetic tree was constructed by the neighbor-joining method with MEGA software. Bootstrap analysis was performed with 100 replicates. The GenBank accession numbers for the 16S rRNA gene sequences included are as follows: *Rickettsia japonica*, L36213; *R. rickettsii*, L36217; *Rickettsia* sp. RpA4, AF120026; *R. aeschlimannii*, U74757; *R. africae*, L36098; *R. conorii*, AE008647; *R. honei*, AF060705; *R. montana*, U11016; *R. parkeri*, U12461; *R. sibirica*, D38628; *R. slovaca*, L36224; *R. helvetica*, L36212; *R. australis*, U12459; *R. akari*, L36099; *R. typhi*, U12463; and *R. prowazekii,* M21789. The GenBank accession numbers for the *gltA* gene sequences of these bacteria are U59724, U59729, AF120029, U59722, U59733, U59730, U59726, U74756, U59732, U59734, U59725, U59723, U59718, U59717, U59714, and M17149, respectively.

Amplification and sequencing of the 16S rRNA, *gltA*, *ompA*, *ompB*, and *sca4* genes were as described by Lee et al. (*6*). Sequences were aligned by using the multiple-alignment algorithm in the MegAlign software package (Windows version 3.12e; DNASTAR, Madison, WI, USA), and phylogenetic trees were constructed by the neighbor-joining method with the MEGA program (*8*). Sequences were compared to the sequences with those of 16 reference strains of SFG rickettsiae (*9**–**11*). The nucleotide sequence (AY743328) of the 16S rRNA gene was identical to that of *R. japonica* YH. Inha1 demonstrated 16S rRNA sequence similarities of 95.9%–99.7% to the other strains of SFG rickettsiae. In the phylogenetic tree, Inha1 formed a cluster with *R. japonica* YH, separate from the other strains of SFG rickettsiae (Figure 2). The nucleotide sequence (AY743327) of the *gltA* gene of Inha1 showed a high similarity (99.8%) with that of *R. japonica* YH. The sequence similarities of Inha1 to the other strains of SFG rickettsiae were 93.6%–99.1%.

The nucleotide sequence of the *ompA*, *ompB*, and *sca4* of Inha1 strain also showed a high similarity to *R. japonica* YH and the sequence similarities to *R. japonica* YH were 100, 99.9, and 99.9%, respectively.

## Conclusions

To identify the isolate at species level, we determined the sequence of five genes that have been used for the phylogenetic classification of rickettsiae (*9**–**14*). The sequences are identical or highly homologous to those of *R. japonica*. Although the sequence similarity of the *gltA* gene of Inha1 strain to *R. japonica* YH was 99.8%, the other 4 genes show sufficient similarity to fulfill the criteria suggested by Fournier et al. (*9*).

*R. japonica* was first isolated in Japan from human patients and ticks (*15*). The isolation of *R. japonica* in South Korea is not surprising because of the geographic proximity of South Korea to Japan. Furthermore, among 4 SFG rickettsiae detected in Korean ticks, 3 strains were highly homologous to *R. japonica* (*6*). Therefore, *R. japonica* may be the most dominant SFG rickettsiae distributed in South Korea, and the geographic distribution of *R. japonica* may be more widespread than previously known. However, other SFG rickettsiae, including *R. sibirica*, may be present in northeastern Asia. To clarify this issue, more strains of SFG rickettsiae must be isolated from other locations within SouthKorea.
